# The Need for School Nursing in Spain: A Mixed Methods Study

**DOI:** 10.3390/ijerph15112367

**Published:** 2018-10-26

**Authors:** Julián Rodríguez-Almagro, Antonio Hernández-Martínez, Gema Alarcón-Alarcón, Nuria Infante-Torres, Miriam Donate-Manzanares, Juan Gόmez-Salgado

**Affiliations:** 1 Deparment of Emergency, Ciudad Real University Hospital, 13005 Ciudad Real, Spain; julianj.rodriguez@uclm.es; 2 Midwife Unit, Mancha-Centro Hospital, 13600 Alcázar de San Juan, Spain; 3 Ciudad Real Ministry of Education Provincial Directorate, 13001 Ciudad Real, Spain; rekete82@hotmail.com; 4 Obstetrics Service, Gutierrez Ortega Hospital, 13300 Valdepeñas, Spain; nuria.i.t@hotmail.com; 5 Obstetrics Service, Gregorio Marañon General University Hospital, 28009 Madrid, Spain; m_donate_manzanares@hotmail.com; 6 Department of Nursing, Faculty of Nursing, University of Huelva, 21071 Huelva, Spain; jgsalgad@gmail.com; 7 Espíritu Santo University, 092301 Guayaquil, Ecuador

**Keywords:** students, health problems, life threatening, teachers, school nursing

## Abstract

*Background*: Teachers are not trained or feel prepared for urgent action. Nevertheless, the presence of children with health problems is relevant. We identified vital health risk problems and complications among students, as well as the related training and perception of teachers. *Methods*: An explanatory sequential design was employed. The study sample consisted of a cross-sectional study of an intentional nonprobabilistic sample of 3246 teachers in the quantitative phase, and a total of 16 semistructured interviews were conducted in its qualitative phase. *Results*: 56.6% (1837) of teachers show high concern about facing such situations and only 0.6% (19) feel appropriately trained. For 81.8% (2556), the existence of school nursing would be quite relevant. The presence of nursing professionals in schools could lead to an improvement in the quality of life of both the students and teachers. *Conclusions*: There is a significant percentage of children with diseases that often require specific care and there is a high probability that teachers, throughout their professional lives, have to deal with situations of vital urgency. The presence of professionals in educational centres seems to be a relevant option. These data suggest that it is necessary for nurses to establish a pilot programme for the incorporation of professional nurses in educational centres to determine its implications, benefits in health prevention and promotion issues, as well as costs.

## 1. Introduction

It is estimated that around 15% of schoolchildren have a chronic health issue [1,2] and that, due to these problems, they can present some degree of inhibition for full integration in their school activities, giving rise to behavioral problems [3]. Generally, the school population has increasingly more chronic pathologies and more deficiencies in health issues. In addition, there is a progressive increase in the number of foreign students with different pathologies and in situations of economic and social marginalization. For all these reasons, the training of health professionals requires greater specialization and adaptation [3], taking into account that there is an undocumented need for giving more specialized attention within different educational settings to the problems that arise in paediatric populations with associated chronic pathologies.

In response to these new health demands, having an in-school nurse has been considered as an interesting option for those responsible for both the healthcare and education sectors. The nurse could play the role of health agent, either from the primary care or educational centre position, being a professional with full responsibility and potential in terms of education and health promotion development in the school community, in addition to acting as a reducing factor of stress and anxiety [4,5,6].

The figure of the school nurse has been implemented in some countries such as France, the United Kingdom, Germany, and Switzerland in Europe and has been fully established in the United States for many years [7]. However, in Spain, a school nurse is only present in some autonomous communities and in ad hoc situations.

For this reason, our aim is to identify vital risk health issues and complications among students as well as the teachers’ training on and identification of the same.

## 2. Materials and Methods

This is an explanatory sequential design in two stages and our aim is to use qualitative data to help us explain the quantitative results initially obtained so as to better develop the quantitative results [8,9]. An explanatory sequential design is a mixed method.

Mixed methods research is increasingly being used as a methodology in the sciences to gain a more complete understanding of issues and hear the voices of participants [10,11]. Mixed methods research is the collection and analysis of both qualitative and quantitative data and its integration, drawing on the strengths of both approaches [12,13] as a way to represent and facilitate the integration of qualitative and quantitative data in mixed methods studies [14].

Within these studies, preliminary quantitative results are used to report on the phenomenological approach within the second phase of the research. Therefore, quantitative data either provide guidance towards the phenomena discovered within the preliminary quantitative phase or help identify participants for the phenomenological phase which can provide information on the experiences of respondents [15,16,17,18,19,20,21,22].

### 2.1. Quantitative Phase Methodology

#### 2.1.1. Design and Scope of the Study

This was an observational, descriptive, and cross-sectional study on an intentional nonprobabilistic sample of 3246 teachers both from general education centres (preschool, primary, secondary, high school diploma, vocational training, special education, and distance learning) and special regime systems (school of plastic arts and design, school of music and dance, official language schools, drama schools, and sports schools) in Spain. The recruitment of participants was conducted between January and March 2017. Only teachers over 65 were excluded.

#### 2.1.2. Estimation of Sample Size

The set of teachers registered in the Ministry of Education of Spain was taken into account for the calculation of the sample size, with a total of 695,598 teachers (EducaBase) in the school year 2016–2017. Fifty percent were used as the reference prevalence, as it was a multiple choice questionnaire and this was the most demanding prevalence. The confidence level was 95%, with an absolute error of 2%, resulting in a minimum of 2393 participants.

#### 2.1.3. Sources of Information

An ad hoc data collection notebook was designed for this purpose. It included an explanation of the aims and purposes of the study and ensured anonymity, confidentiality, and further ethical guarantees. This questionnaire (Appendix A) was sent to various associations or groups of teachers through social networks as well as to the various provincial delegations of education which, in turn, referred this questionnaire to all educational centres, and the contribution of the regional teaching institution was especially important for the task of diffusion distribution of the questionnaire to all centres and responsible teachers in a systematic manner.

#### 2.1.4. Study Variables

The questionnaire collected information on sociodemographic variables about the professional profile of teachers, their experience in students’ health risk situations, the existence of children with health problems in the current academic year, the degree of training for and concern over these situations, as well as the relevance of the hypothetical figure of the school nurse.

#### 2.1.5. Statistical Analysis

First, descriptive statistical analysis using absolute and relative frequencies for the qualitative variables was conducted.

For the bivariate analysis, Pearson’s chi-squared test was used when both variables were qualitative, and the Mann-Whitney U nonparametric test was used when studying the relationship between the variables with ordinal response and qualitative dichotomous ones.

All analyses were performed with the statistical package SPSS 24.0 (SPSS Inc., Chicago, IL, USA).

#### 2.1.6. Ethical Aspects

This study was designed in accordance with the Helsinki Declaration adopted by the World Medical Association (WMA). The completion of the questionnaire was completely voluntary and anonymous.

Approval by the Ethics in Research Committee Mancha Centro Hospital with number C-149 February 2017 was granted before starting the research. 

### 2.2. Qualitative Phase Methodology

A descriptive, qualitative, phenomenological design based on Giorgi’s method [23] was developed. It was aimed at teachers and its information analysis was carried out using the phenomenological method of Amedeo Giorgi [24].

This was a purposive sampling [25], conducting interviews with those teachers who were in contact with the research team after having seen the appeals made in social networks during the months of January–March 2017, and although the theoretical sample size was insufficient to guarantee external validity in terms of other models of research [26], it was sufficient to complete all categories, including participants from different sociodemographic characteristics.

The collection of data through interviews was recorded on audio files with a password, and only the authors had access to the same [27].

The only criterion for inclusion was being a teacher with an average age of 21–65 years.

A total of 16 semistructured interviews were conducted, collecting a number of sociodemographic characteristics such as age or sex following a semistructured script we created from the quantitative phase to modulate the interviews, with a duration of between 40 and 60 min, which were transcribed in their entirety. The participants did so voluntarily and with informed consent. The teachers were identified by codes to respect their anonymity, identifying discourses with the letter E (for “interview” in its Spanish form entrevista), followed by a sequential number from 1 to 16.

All interviews began with an open question to invite participants to narrate their experiences as teachers and the need for a nurse in their centres so that they could focus on the phenomenon in question [23]: Could you tell me what it is for you to be a teacher and if you think it is necessary to have a nurse at educational centres? Participants were encouraged to freely narrate their experience, and the interviewer would freely follow his script to encourage them.

The credibility, auditability, and transferability methodological rigour criteria [28] were taken into account during the process.

## 3. Results

### 3.1. Quantitative Phase Results

This work involved a total of 3246 teachers in Spain, of which 65.8% (2136) were women, and the most numerous age group consisted of teachers aged between 31 and 40 years (34.8% (1131)). 74.3% (2409) had been teaching for more than 10 years. Further information can be found in Table 1.

As to whether teachers had received specific training in urgent action on children’s health problems, 54.9% (1783) had never participated in such training, 13.2% (429) had received training in the last 5–10 years, 21.0% (683) in the last 1–5 years, and 10.8% (351) in the last year.

In this sense, the relationship between the training received and the profile of teachers was analysed, observing a statistically significant association with the age of teachers and the years of practice (*p* < 0.001). In the light of this, teachers who were older and had more years of practice had a higher percentage of training conducted. However, from 50 years of age and with more than 20 years of teaching, there was a decline in the amount of training. It should also be noted that, regardless of these results, there is a percentage higher than 50% in all categories of teachers who have never received this training (Table 1).

Then, teachers were asked about whether, in their professional career, they had experienced a series of health problems requiring urgent action. To this question, the most frequently observed problems were dizziness and/or syncope with 75.4% (2447), followed by injuries requiring sutures or plaster splints with 61.2% (1986), and hypoglycaemia with 52.2% (1693).

In addition, teachers were asked about their level of concern over the occurrence of any of these health problems, and we found that 56.6% (1837) of teachers were very concerned about it and 33.0% (1071) were fairly concerned (Table 1).

They were also asked to what extent they were prepared to face any of these problems, of which 16.4% (533) answered they were not prepared and 46.9% (1523) expressed feeling scarcely prepared (Table 1).

In this case, relating the degree of concern and the perception of their preparedness with the completion of training in urgent action, we found a statistically significant association. Teachers who had received training had increased their perception of preparedness (*p* < 0.001) and concern (*p* = 0.027) with respect to those not trained.

Then, so as to have an overall view of the health state of children and adolescents attending educational centres in Spain, teachers were asked if there was at least one child with a series of health issues in their classrooms. Results can be found in Table 2, which highlight that 69.6% (2258) of teachers had students with attention-deficit hyperactivity disorder in their classrooms, 62.8% (2038) with food allergies, and 56.8% (1845) with asthma.

Finally, when asked to what extent could the presence of a specialized nurse be relevant in educational centres to deal with these situations, 51.9% (1684) stated that it would be very important and 29.9% (972) quite relevant (Table 1). In this sense, the trained teachers give greater relevance to the figure of the school nurse than those who are not trained (*p* = 0.001).

### 3.2. Qualitative Phase Results

#### 3.2.1. Participants

The analysis of the interviews allowed us to stablish four categories that are representative of the life experience of teachers and the need to have a school nurse in educational centres. These categories have been named as: Need when facing reality, Fears, Responsibility, and Limitations as a teacher (Table 3).

#### 3.2.2. Need When Facing Reality

Our study teachers felt the need for a nurse in educational centres, but at the same time, there was a feeling of disappointment in terms of their reality regarding the need for teachers. Therefore, on the one hand, they believe it necessary to have a nurse in their centres, but surely not at the expense of reducing their own staff or hiring fewer teachers in order to have a nurse in each centre. There is a clear dilemma and the need for an unfounded choice between having a school nurse and hiring more teachers:

“*It would be great, but it would be better to have the necessary number of teachers.*”

(E3)

“*I believe that a nurse by centre should be mandatory, but more teachers would also be good.*”

(E5)

“*It would be great! But in the light of how things are usually done, they could even hire a nurse by centre and tell them to be substitute teachers.*”

(E9)

#### 3.2.3. Fears

From the interviews, we obtained data regarding being a teacher in charge of taking caring of children with chronic diseases; this fact leads them to have a number of fears based on the lack of knowledge about what to do in certain cases which can occur throughout their professional career:

“*To be honest, a nurse is quite necessary in our schools and high schools. I have found myself, more than once, due to my specialty, in the situation of students with deep cuts, and it feels really bad. You don’t really know what to do, and with serious mishaps we have to perform a quick cure without any means (I teach cooking and pastry, and in many centres, there is no first-aid kit), and we need to go to the hospital in a rush...Kids with epilepsy, diabetes, cardiac problems.*”

(E6)

“*I have a student with epilepsy and am always afraid of something bad happening. Even with indications of how to intervene, I don’t know if I would be able to do it properly. I am completely in favour of a nurse in every school.*”

(E8)

#### 3.2.4. Responsibility

In the data reduction, the term “responsibility” recurrently emerged. Teachers believe that assisting a student is not their responsibility, since it implies burdens they do not want to carry and are reluctant to do so; they expressed the need of a school nurse, although their words carry a number of nuances, such as considering the omission of assistance a crime.

“*I would also like it if there were a nurse in every educational centre, and even more after knowing that, in case of emergency, we will have to inject what they need (if their life is in danger) and if we don’t, it is a crime.*”

(E7)

“*It is crime, and it can also be the ruin of you if the judge believes that you did not act as the parents would have done. Not to mention if you rush to seek help...They can tell you you’ve abandoned the student.*”

(E5)

As an emerging expectation in all the speeches, the concerns about the limitations they have in relation to their work and way of acting arise. Teachers see the need for training in first-aid courses, although they continue to think there is the need for a school nurse:

“*Well, so they should then require a first-aid course as a minimum. Rather than scoring online courses taken just because, they should score first aid. I sincerely believe this is a limitation.*”

(E11)

“*We have taken the trouble to take first-aid courses, but it would be better to have a qualified person to do so.*”

(E15)

## 4. Discussion

In the present study, the opinions of teachers regarding their perception of the degree of training and concern in first aid with children was evaluated, as well as the need for a nurse in educational centres of Spain.

Regarding this, it is highlighted that more than half of the teachers have not received any training, with percentages of high concern of 56.6% and with a perception of deficient training to deal with these problems of 46.9%. Hence, the presence of a nurse at educational centres was very positively valued. Based on these results during the qualitative process, four broad categories have been set: need when facing reality, fears, responsibilities, and limitations as a teacher.

On the other hand, we can highlight that up to 75.4% of the teachers have witnessed dizziness or syncope, and 61.2% have witnessed trauma in children that have required stitches or plasters, concurring with other studies [29,30]. We must here add that 69.6% of teachers declare having a student with chronic problems. This situation creates anxiety for some teachers, as is highlighted in the qualitative results that coincide with other authors such as Gomez [31] and Fernandez [32]. These refer to teachers who acknowledge having or having had a student with a chronic pathology, and this issue causes them some degree of uncertainty.

These situations have as a consequence that the incorporation of nurses in schools is not only perceived as relevant by teachers, but that, as other authors such as Calvo [33] and Díez [34] have found, between 72% and 86% of parents, respectively, claim that the presence of a nurse at the educational centre is necessary.

The limitations of our study include those arising from the absence of a nonrandom sampling procedure. The attitudes of the people who answered voluntarily could have been more favourable than that of those who did not participate. It is possible that the participation in this study reflects a greater involvement of teachers in need of a school nurse or just the opposite. Despite this, we believe that it is a quite accurate portrait of reality because the qualitative part of the study confirms that teachers feel there is a lack of knowledge and training in health education, although some of them allege that nurses should be the ones having training in schools, and that the responsibility should not fall on them as teachers.

### Implications for Nursing and Health Policy

Nevertheless, an alternative to the school nurse could be developed through an ambitious training and sensitization plan for teachers. However, these plans could not guarantee that all teachers are trained, as can be seen in our results, and, on the other hand, it is difficult to achieve the appropriate capacity for technical aspects such as the preparation of medication and its administration [35]. In addition, the school nurse would not only be limited to resolving specific mishaps, but they would have a more ambitious role in the field of promotion and prevention. Duties would range from a closer monitoring programme of healthy children to teaching in seminars both for teachers and students, and even the application of cures or specific techniques at schools that would prevent school absenteeism due to the transfer to and from the health centres, etc. This aspect is particularly important when taking into account the results observed in our study on the frequency of children with pathologies. It would be interesting to develop a pilot project to assess the impact that the presence of school nurses at all levels has on: public health, occupational risk prevention, reducing hospital admissions, school absenteeism, prevention of risk behaviours, occupational quality of teachers, anxiety in teachers and parents, and even, in economic terms, a training plan for teachers in this subject or any other alternative.

To incorporate a nurse into Spanish schools at present, it would only be necessary for the political leaders in the management of the educational centres to be convinced of the importance of the creation of this figure. In Spain, there are nursing professionals with specific degrees in community nursing and paediatric nursing who could assume this role. On the other hand, the creation of this new figure could result in savings for the health system, as other studies have shown [36]. The implementation of the teaching nurse should be progressive. Initially, pilot experiences should be developed in different centres before their systematic implementation to establish the functions of the teaching nurse and the evaluation of their economic impact.

## 5. Conclusions

As a conclusion, we find that there is a significant percentage of children with diseases that often require specific care and that there is a high probability that teachers, throughout their professional lives, have to deal with situations of vital urgency. In this sense, teachers in Spain are not trained in urgent action. In addition, they have the perception of having low training and this is an important concern. The presence of professionals in educational centres seems a relevant option, although they manifest a dilemma regarding the fear that these professionals may imply a decrease of teaching professionals.

## Figures and Tables

**Table 1:** Relation between teacher profile and training received.

Characteristic	Total	Training Received		*p*-Value
	(*n*/%)	No (*n*/%)	Yes (*n*/%)	
Sex				0.051 ^a^
Male	1110 (34.2)	636 (57.3)	474 (42.7)	
Female	2136 (65.8)	1147 (53.7)	989 (46.3)	
Age				0.001 ^a^
<30	205 (6.3)	137 (66.8)	68 (33.2)	
31–40	1131 (34.8)	606 (53.6)	525 (46.4)	
41–50	1121 (34.5)	592 (52.8)	529 (47.2)	
>50	789 (24.3)	448 (56.8)	341(43.2)	
Years as a teacher				<0.001 ^a^
<1 year	96 (3.0)	73 (76.0)	23 (24.0)	
1–5 years	275 (8.5)	184 (66.9)	91 (33.1)	
6–10 years	466 (14.4)	263 (56.4)	203 (43.6)	
11–15 years	775 (23.9)	405 (52.3)	370 (47.7)	
16–20 years	620 (19.1)	306 (49.4)	314 (50.6)	
21–30 years	716 (22.1)	395 (55.2)	321 (44.8)	
>30 years	298 (9.2)	157 (52.7)	141 (47.3)	
Degree of concern				0.027 ^b^
High	1837 (56.6)	980 (55.0)	857 (58.6)	
Quite high	1071 (33.0)	603 (33.8)	437 (32.0)	
Medium	244 (7.5)	147 (8.2)	97 (6.6)	
Low	85 (2.6)	46 (2.6)	39 (2.7)	
No concern	9 (0.3)	7 (0.4)	2 (0.1)	
Degree of training				<0.001 ^b^
High	19 (0.6)	6 (0.3)	13 (0.9)	
Quite high	151 (4.7)	34 (1.9)	117 (8.0)	
Medium	1020 (31.4)	423 (23.7)	597 (40.8)	
Low	1523 (46.9)	903 (50.6)	620 (42.4)	
No training	533 (16.4)	417 (23.4)	116 (7.9)	
Need for school nurse				0.001 ^b^
High	1684 (51.9)	980 (53.3)	857 (46.7)	
Quite high	972 (29.9)	603 (56.3)	437 (47.5)	
Medium	356 (11.0)	147 (60.2)	97 (39.8)	
Low	174 (5.4)	46 (54.1)	39 (45.9)	
No need	60 (1.8)	980 (53.3)	857 (46.7)	

^a^ Pearson’s chi-squared test; ^b^ Mann-Whitney U test.

**Table 2:** Students health issues in the current course, as notified by teachers.

Issue	(*n*/%)
Metabolic disorders	337 (10.4)
Heart problems	658 (20.3)
Epilepsy	671 (20.7)
Serious neurological problems	1051 (32.4)
Diabetes	1076 (33.1)
Atopic dermatitis	1206 (37.2)
Obesity	1382 (42.6)
Psychiatric disorders	1413 (43.5)
Celiac disease	1416 (43.6)
Asthma	1845 (56.8)
Food allergy	2038 (62.8)
Attention-deficit hyperactivity disorder	2258 (69.6)

**Table 3:** Sociodemographic characteristics of teacher (qualitative phase).

Interview *n* = 16	Age	Time Working as a Teacher
1 (Male)	24	1 Year
2 (Female)	22	1 Year
3 (Female)	52	20 Years
4 (Male)	33	5 Years
5 (Male)	47	15 Years
6 (Male)	28	3 Years
7 (Female)	41	11 Years
8 (Female)	49	22 Years
9 (Male)	33	6 Years
10 (Female)	41	14 Years
11 (Female)	25	2 Years
12 (Female)	60	30 Years
13 (Male)	43	7 Years
14 (Male)	62	32 Years
15 (Female)	30	7 Years
16 (Male)	36	5 Years

## Appendix A. Questionnaire on the Concerns Expressed by Teachers about the Appearance of Health Problems in Their Students

1. Age (years):
2. Sex
  Man
  Woman
3. How many years have you been practicing as a teacher (approximately)?
4. Course to which he teaches his classes at present:
5. Have you participated in any specific training on urgent action in the face of a health problem in children?
  No
  Yes
6. If yes, when was the last time?
  In the last year
  Between 1 and 5 years
  Between 5 and 10 years
7. Mark with a cross if you have witnessed any of these problems in the children in your class.
  Cardiorespiratory arrest
  Allergic reaction
  Hypoglycaemia (low blood sugar)
  Epileptic seizures
  Trauma that requires suture
  Dizziness or syncope
  Choking
8. To what extent do you worry that any of these health problems may occur in your presence?
  Nothing
  Little bit
  Something
  Quite
  Much
9. To what extent are you prepared to face any of these health problems?
  Nothing
  Little bit
  Something
  Quite
  Much
10. Mark with a cross if you currently have a child in your class with these health problems
  Obesity
  Atopic dermatitis
  Heart problems
  Diabetes
  Celiac disease (gluten intolerance)
  Epileptic
  Food Allergy
  Asthma
  TDH
  Metabolic disease (hypothyroidism, phenylketonuria, etc.)
  Neurological disease (For example: cerebral palsy)
  Other psychiatric problems (autism, depression, etc.)
11. To what extent do you consider that the presence of a specialized nurse professional could be relevant to deal with the emergency situations and health problems indicated above?
  Nothing
  Little bit
  Something
  Quite
  Much
